# The Effect of Escin on the Plasma Membrane of Human Red Blood Cells

**DOI:** 10.3390/ijms26188923

**Published:** 2025-09-13

**Authors:** Lukasz Gwozdzinski, Anna Pieniazek, Krzysztof Gwozdzinski

**Affiliations:** 1 Department of Pharmacology and Toxicology, Medical University of Lodz, 90-752 Lodz, Poland; 2 Department of Oncobiology and Epigenetics, Faculty of Biology and Environmental Protection, University of Lodz, 90-236 Lodz, Poland

**Keywords:** escin, red blood cells, membrane fluidity, proteins and cytoskeleton proteins

## Abstract

Escin is a steroidal triterpene saponin isolated from the seeds of horse chestnut (*Aesculus hippocastanum* L.). Due to its anti-edematous, anti-inflammatory, and venotonic properties, it is used in the treatment of chronic venous insufficiency. This study aimed to determine the effect of escin on human red blood cells (RBCs). The effect of escin on RBC hemolysis, plasma membrane fluidity, and thiol, amino, and carbonyl group levels was examined, while the conformational state of membrane proteins was also determined. Low concentrations of saponin (15–60 µg/mL) led to RBC hemolysis and increased RBC membrane fluidity, as determined using the spin probe method. Escin caused a statistically insignificant increase in thiol groups but a significant increase in amino and carbonyl groups in cell membranes. Using two spin labels that covalently bonded with thiols, we demonstrated that treatment of RBCs with escin did not affect cytoskeletal proteins or plasma membrane surface proteins. Research indicates that the main target of escin’s action is the lipid portion of the membrane, not membrane proteins.

## 1. Introduction

β-Escin is a saponin, a biologically active component of horse chestnut seeds (*Aesculus hippocastanum*). Horse chestnut saponins have a similar structure, consisting of a triterpene or steroid hydrophobic backbone and containing varying numbers of sugar residues [1,2]. Saponins consist of an aglycone (the hydrophobic part of the molecule), to which oligosaccharides are attached. Horse chestnut extract contains approximately 30 different molecules, of which β-escin constitutes approximately 60% [3]. In the case of β-escin, the triterpene backbone is linked via glucuronic acid to two glucose units. Compared to α-escin, the β isomer (β-escin) is characterized by significantly higher pharmacological activity [4,5,6].

Research conducted on over 1700 varieties of Asian plants and vegetables has shown that saponins occur in 75% of them [7]. Despite the significant differences in the structure of these compounds, their common feature is the presence of a steroid or triterpenoid backbone (aglycone) [8]. Typically, the 3-position of the backbone contains glucuronic acid and sugar residues such as glucose (Glc), xylose (Xyl), and galactose (Gal). Such structures also occur in various escins. A common feature of escins is the presence of a tigloyl and angloyl substituent [3,9]. The extract obtained from horse chestnut (*Aesculus hippocastanum*) may contain 97.0–100.0% escins, which are a mixture of triterpene saponins composed mainly of escin Ia, escin Ib (β-escin), and isoescin Ia and isoescin Ib (α-escin) [10].

**Figure 1:**
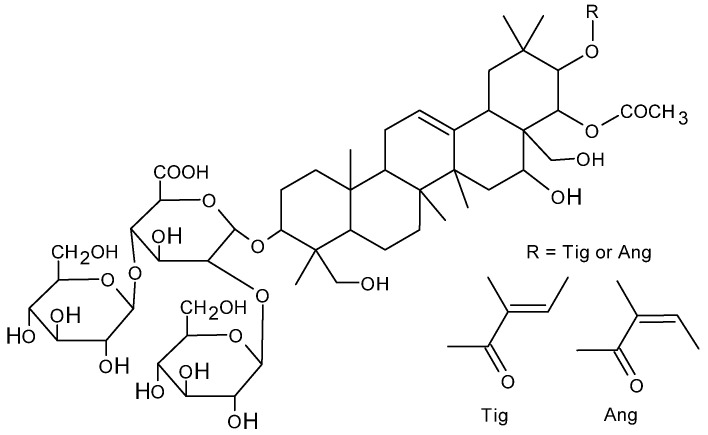
Chemical structure of β-escin.

β-escins can have different forms. Escin Ia has a tigloyl substituent (Tig) (cis isomer) at the C21 position of the triterpene ring and an acyl group at the C22 position, while escin Ib has an angloyl substituent (Ang) at the 21 position (trans isomer) and also an acyl group at the C22 position (Figure 1).

Escin IIa has the same substituents at the C21 and C22 positions but differs in its a sugar residue at the 2′ position of glucuronic acid because xylose is present instead of glucose [11]. Unlike β-escins, α-escins (isoescins) have acyl groups at the C28 position instead of hydrogen atoms [4,5]. In the α-escin group, there are also cis and trans geometric isomers. It has been shown that the basic forms of escin, i.e., α and β, differ in their molecular weight and differ significantly in their melting point, specific rotation, hemolytic properties, and water solubility [4].

Saponins found in many plants have interesting pharmacological properties, which is why they have been used since ancient times to treat various diseases. Escin has anti-inflammatory, anti-edematous, and venotonic properties [4,12,13]. It is also active against various types of cancer, such as lung adenocarcinoma, hepatocellular carcinoma, ovarian cancer cells, and leukemia cell models [14,15]. The main use of escin is in the treatment of post-thrombotic syndrome and chronic venous insufficiency [16,17,18,19]. Furthermore, escin has been shown to have anti-inflammatory effects in the treatment of arthritis and also anticancer effects [11,14,20].

Plant extracts, frequently used in herbal medicine, demonstrate stronger activity than isolated biologically active substances. The explanation lies in the synergistic effects of various combinations of active compounds. It is known that lipophilic monoterpenes or saponins are characterized by a targeted effect on cell membranes. For example, the combination of escin with thymol demonstrated approximately 100 times more potent hemolytic activity than escin alone. A similar situation occurs with saponins accompanied by polyphenols or other biologically active substances. Such combinations can also be expected to exhibit synergistic effects [21]. For this reason, various drug combinations are sometimes used in the treatment of chronic venous disease. Horse chestnut saponins belong to a group of natural medicines called phlebotonics. These medicines are used in the treatment of venous insufficiency and thrombophlebitis, primarily varicose veins, leg ulcers, and hemorrhoids [4]. They are considered safe drugs that can be administered orally or topically. They have anti-inflammatory, anti-edematous, and venous-relieving properties [4,22].

Saponins interact with artificial phospholipid membranes and cell membranes, including red blood cells (RBCs), cancer cells, and others, leading to changes in their structure and properties [3,21,23,24]. It has been shown that β-escin interacts with DMPC vesicles (1,2-dimyristoyl-sn-glycero-3-phosphocholine bilayer vesicles) differently depending on its concentration. Incorporation of β-escin into the DMPC bilayer leads to increased bilayer fluidity. However, with an increasing β-escin concentration, the vesicles become unstable in solution and form agglomerates [23]. In another study, external incorporation of escin induced membrane stiffening in DMPC bilayer vesicles [25]. Although phenolic derivatives are the most common natural compounds, saponins are produced by over 50% of plants [26,27]. It has been shown that the lipophilic fragment of saponins forms complexes with cholesterol, while the hydrophilic fragment interacts with membrane proteins, altering their conformational state, which consequently leads to changes in the fluidity of the cell membrane [28,29]. However, at higher concentrations, saponins can lead to lysis of plasma membranes. Some saponins have selective cytotoxic effects on cancer cells, but their hemolytic properties reduce their anticancer potential [24,30]. Escin has been shown to cause hemolysis of RBCs and to be cytotoxic to HeLa and Cos7 cells. Both cancer cell lines were approximately 10 times more sensitive than RBCs [21].

In this study, we investigated the effects of escin on human red blood cells, focusing primarily on the properties of the RBCs’ plasma membranes. The degree of RBC hemolysis; the fluidity of membrane lipids; the conformational state of membrane proteins; and the levels of thiols, amino groups, and carbonyl groups were determined.

## 2. Results

**Figure 2:**
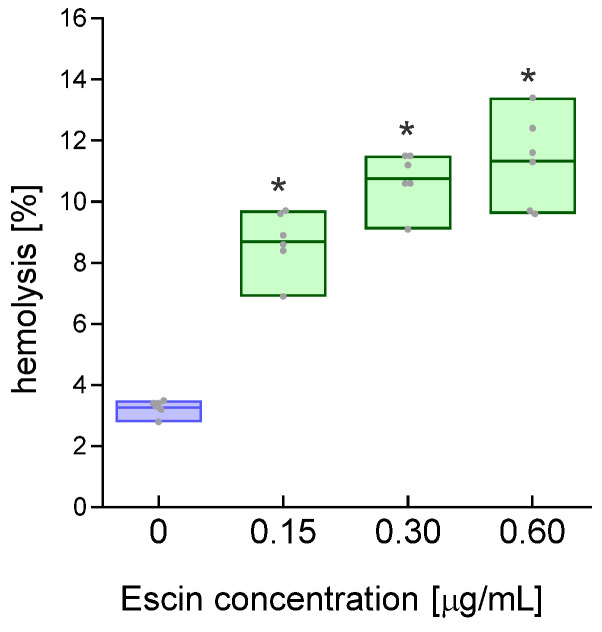
Hemolytic effect of escin on RBCs after 24 h of treatment. The data were expressed as mean with bars (min to max) and raw data, n = 7. Statistical significance: * *p* < 0.05 vs. control.

Escin from the horse chestnut tree is a natural surfactant that is widely used in pharmacology. In our study, we examined the effects of this compound on the properties of red blood cell membranes when exposed to it. Isolated RBCs were exposed to escin at three selected concentrations (0.15, 0.30, and 0.60 µg/mL) for 24 h. Studies have shown that this compound causes a significant increase in the osmotic fragility of RBCs in a solution of 155 mM NaCl (Figure 2). Escin at a concentration of 0.15 µg/mL caused more than 2.5 times higher hemolysis compared to the values obtained for control cells. The percentage of hemolyzed cells increased with the concentration of escin. At a concentration of 0.60 µg/mL, escin increased the osmotic fragility of erythrocytes approximately 3.5 times compared to controls, resulting in hemolysis of more than 11% of the cells.

Given that the cell membrane consists principally of proteins and lipids, it appeared crucial to assess the status of these components. The use of doxyl derivatives of fatty acids incorporated into the outer layer of the cell membrane in EPR spectroscopy provides information about the fluidity in the surroundings of the spin probe. The fluidity of the outer monolayer of the cell membrane was studied at three depths of the fatty acid hydrocarbon chains. The unpaired electron in 5-doxyl-stearic acid reflects changes in fluidity in the polar parts of the membrane, while 12-doxyl-stearic acid and 16-doxyl-stearic acid indicate changes in the hydrophobic regions of the cell membrane.

**Figure 3:**
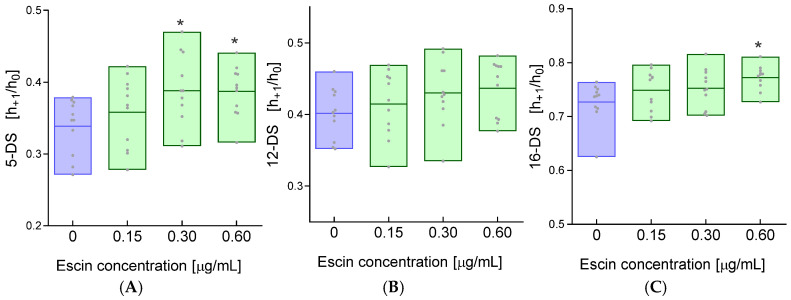
Changes in the h_+1_/h_0_ ratio for the (**A**) 5DS, (**B**) 12DS, and (**C**) 16DS spin labels incorporated into the cell membrane of RBCs treated with escin. The data were expressed as mean with bars (min to max) and raw data, n = 11. Statistical significance: * *p* < 0.05 vs. control.

For the 5DS spin probe, which has an unpaired electron located close to the membrane surface, a significant increase in the h_+1_/h_0_ ratio was observed after incubation of erythrocytes with escin at concentrations of 0.30 and 0.60 μg/mL compared to the values obtained for control cells (Figure 3A).

For the 12DS probe incorporated into the cell membrane of RBCs treated with escin, no statistically significant changes in the h_+1_/h_0_ ratio were observed (Figure 3B). At a depth of 16 carbon atoms of fatty acids, a significant increase in the h_+1_/h_0_ ratio was observed in erythrocytes treated with escin at a concentration of 0.60 μg/mL compared to control values. In erythrocytes incubated with lower concentrations of escin (0.15 and 0.30 μg/mL), changes in membrane fluidity for the 16DS probe were statistically insignificant (Figure 3C). However, a significant increase in the h_+1_/h_0_ ratio was observed at the highest escin concentration (0.60 µg/mL). In the EPR spectrum, this is manifested by an increase in the intensity of the low-field line (h_+1_), central line, and high-field lines (h_−1_) of the EPR spectrum. Consequently, the ratio of line height h_+1_ to line height h_0_ increases, reflecting increased cell membrane fluidity.

**Figure 4:**
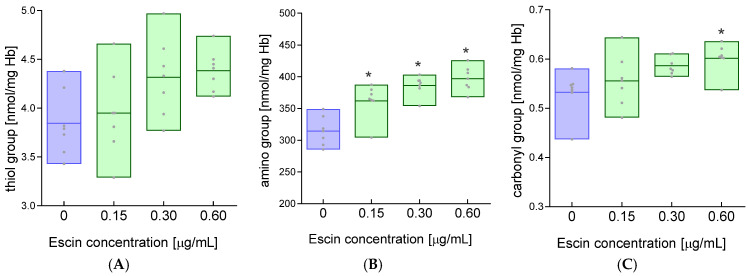
Changes in the level of (**A**) thiol, (**B**) amino, and (**C**) carbonyl groups in membrane proteins after incubation of RBCs with escin. The data were expressed as mean with bars (min to max) and raw data, n = 7. Statistical significance: * *p* < 0.05 vs. control.

A factor influencing normal cell membrane function is the state of membrane proteins. Amino acid modifications in proteins can lead to changes in the second-, third-, and fourth-order structure, which sometimes have functional consequences. We investigated the level of basic functional groups in erythrocyte membrane proteins after incubation with escin. The study showed a slight increase in the level of thiol groups in erythrocyte membrane proteins after their incubation with escin (Figure 4A) and at the same time significantly increased levels of amino groups in erythrocyte membranes for all concentrations of escin compared to the values obtained for control cells (Figure 4B). The study also showed a significant increase in the level of carbonyl groups in the membranes of cells incubated with escin at a concentration of 0.60 μg/mL compared to the membranes of control cells (Figure 4C). In contrast, for lower concentrations of escin (0.15 and 0.30 μg/mL), no significant increase in the level of carbonyl groups was observed in erythrocyte membrane proteins compared to controls.

The results obtained for the levels of various functional groups in cell membranes pushed us to evaluate the conformational state of the membrane proteins of escin-treated erythrocytes. Hence, in the next stage of the study, using spin labels (MSL and ISL), we assessed the conformational state of the membrane proteins of erythrocytes treated with escin. The two spin labels (MSL and ISL) selected to study the conformational state of the proteins react selectively with the thiol (-SH) groups of the cysteine residue to form a stable covalent thioether bond. MSL binds mainly (80%) with the inner membrane surface, and >75% of this spin label is attached to the spectrin and actin complex. In turn, ISL reacts with surface proteins of the plasma membrane [31]. The calculated h_+1_/h_0_ ratio is a sensitive indicator of local mobility and dynamics at the spin label attachment site, allowing inferences about the structure, conformation, and interactions of membrane proteins in their native environment.

**Figure 5:**
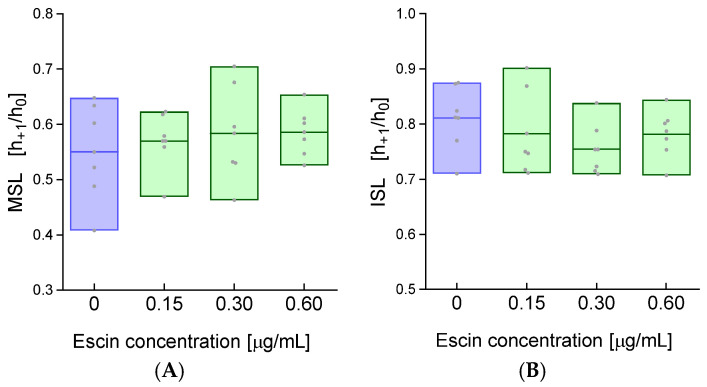
Changes in the conformational state of RBC membrane proteins incubated with escin, measured using two labels: (**A**) MSL and (**B**) ISL. The data were expressed as mean with bars (min to max) and raw data, n = 7.

The study of the conformational state of RBC membrane proteins using the MSL spin label showed insignificant changes in the h_+1_/h_0_ parameter in escin-treated erythrocytes compared to controls (Figure 5A). Similarly, for the ISL spin label bound to membrane proteins of RBCs exposed to escin, no statistically significant changes were observed compared to membrane proteins of control cells (Figure 5B).

## 3. Disscussion

Saponins are composed of a hydrophobic part (aglycone) and oligosaccharide residues (glycone). The hydrophobic part is a pentacyclic terpene, composed of fused six-membered rings, similar to the saponins from *Centella asiatica* and *Ruscus aculeatus* L. (butcher’s broom), but R. aculeatus saponins have three six-membered rings and two five-membered rings [32]. Because not all saponins lead to red blood cell lysis, they are divided into hemolytic and nonhemolytic groups. Membranes are the primary targets for saponins, including escin, and this applies to both artificial lipid membranes and cell membranes. Biological membranes can be described as fluid mosaic structures, where molecules, primarily lipids and proteins, laterally diffuse within the same monolayer of the cell membrane’s lipid bilayer [33]. The fluid mosaic structure allows membrane proteins to diffuse within lipid domains that are suspended in a continuous fluid phase [34]. Cell membranes are characterized by appropriate structural stiffness and lateral fluidity, which allows them to maintain solid and liquid properties to control functional homeostasis [35,36]. Recently, it has been shown that the outer lipid monolayer contains twice as many unsaturated phospholipids as the inner (cytoplasmic) monolayer. Interestingly, the outer monolayer is more densely packed and restricts the diffusion of molecules more than the inner monolayer; this asymmetry is preserved in the endocytic system. The asymmetry of the plasma membrane is revealed in the asymmetric structures of transmembrane protein domains [37]. When interacting with membranes, saponins embed their hydrophobic (lipophilic) moiety into the membrane. In the escin molecule, a hydrophobic moiety is linked to two glucose residues via glucuronic acid. As amphiphilic compounds, inside the membrane, saponins reversibly form pores, which leads to increased membrane permeability to ions and other small molecules. However, the formation of pores is accompanied by membrane disruption, as saponins interact with lipids, primarily cholesterol, forming complexes that lead to increased membrane fluidity [23]. Such interactions can also cause cholesterol extraction from the membrane. This is particularly true for terpene saponins, one of which is escin. This occurs at relatively low concentrations of these compounds, as higher concentrations lead to cell membrane disintegration and lysis [38]. In our studies, we demonstrated that low concentrations (15, 30, and 60 µg/mL) of escin led to a concentration-dependent increase in hemolysis (Figure 1), which is evidence of disintegration of the RBCs’ plasma membranes. RBC hemolysis was also observed in another study using sheep red blood cells with an escin concentration of 316 μm/mL and an incubation time of 30 min [21]. The fluidity of plasma cell membranes is an essential property of cell physiology, determining, among other things, the transport of electrolytes and nonelectrolytes across membranes. Importantly, membrane fluidity changes in various cellular states, under the influence of various substances, including drugs, and in the course of many diseases. Using the spin probe method in EPR spectroscopy, we studied the fluidity of whole red blood cell membranes using three probes positioned at different depths of the outer monolayer of the plasma membrane. We demonstrated that escin at higher concentrations induced an increase in membrane lipid fluidity in the subsurface region of the membrane and the deeper regions of the monolayer at the highest concentration used. Interestingly, we observed no changes in fluidity for the 12DS probe, which localized in regions rich in double bonds found in fatty acids. Membrane fluidity is determined by the amount of unsaturated fatty acids in the lipids, the lipid-to-cholesterol ratio, and the presence of integral and peripheral proteins, as well as protein–lipid interactions and the interaction of the lipid bilayer with the cytoskeleton. The observed increase in membrane fluidity may be due to the interaction of escin with lipids, especially cholesterol, or its extraction from the membrane. Generally, an increase in cholesterol concentration leads to a decrease in membrane fluidity. Furthermore, its presence causes decreased membrane permeability. Changes in membrane structure are also indicated by an increase in thiol groups, although not statistically significant, as well as an increase in amino groups observed for all escin concentrations used. These results may indicate the exposure of these groups during plasma membrane disintegration. On the other hand, a decrease in thiol groups was observed, but with a 1000-fold higher concentration of saponin (300 µg/mL), which was associated with protein aggregation [39]. We also observed modulation of membrane fluidity by escin in endothelial cells [40]. It has also been reported that β-escin strongly stimulated cholesterol synthesis, leading to a significant reduction in the integrity of the actin cytoskeleton in endothelial cells [41].

The increase in carbonyl groups in the membrane at the highest escin concentration is undoubtedly intriguing. Carbonyls are a biomarker of oxidative stress, and escin is an antioxidant that inhibits oxidative stress. These groups were likely previously present in the proteins and were exposed after escin’s action on the membrane. In general, the absence of cholesterol in model bilayers after saponin treatment led to an increase in the anisotropy of fluorescent probes or the order parameter in the case of spin probes. However, in the presence of cholesterol, the lipid anisotropy of fluorescent probes and the order parameters of labeled phospholipids and cholesterol decreased, indicating an increase in membrane fluidity [24]. An increase in membrane fluidity was also observed in DMPC vesicles after exposure to low concentrations of β-escin [23]. Using the spin label MSL, which covalently binds to the thiol groups of the spectrin–actin complex, revealed no changes in the conformational state of these proteins after treatment of RBCs with varying concentrations of escin. These results may indicate that membrane disintegration primarily affects the lipid component. This conclusion is also supported by the lack of changes in the structure of peripheral proteins studied using the ISL label. On the other hand, Bauman and colleagues demonstrated that sublytic concentrations of saponin initiated the aggregation of band 3 protein in the erythrocyte membrane. Saponin-induced hemolysis also affected the interaction between transmembrane proteins and the cytoskeleton but did not lead to cytoskeletal dissociation [39]. These results are consistent with our findings.

## 4. Materials and Methods

### 4.1. Chemicals

The following reagents were obtained from Merck Life Science (an affiliate of Merck KGaA, Darmstadt, Germany): 5,5′-dithiobis(2-nitrobenzoic acid) (DTNB), 2,4,6-trinitrobenzene sulfonic acid (TNBS), 2,4-dinitrophenylhydrazine (DNPH), 4-Maleimido-2,2,6,6,-tetramethylpiperidine-1-oxyl (MSL), 4-iodoacetamide-2,2,6,6, -tetramethylpiperidine-1-oxyl (ISL), 5-doxyl-stearic acid (5DS), 12-doxyl-stearic acid (12DS), 16-doxyl-stearic acid (16DS), and escin (purity ≥ 95%, catalog number: 6805-41-0). Unless stated otherwise, all other chemicals (analytical-grade) were sourced from POCH S.A. (Gliwice, Poland).

### 4.2. Experimental Protocol

The study focused on erythrocytes (RBCs) extracted from the buffy coat of human blood, which was obtained from the blood bank in Lodz, Poland. Pure RBCs were obtained by washing them three times with phosphate-buffered saline (PBS, 10 mM, pH 7.4). To the cells (50% hematocrit in Ringer’s buffer), an aqueous solution of escin was added to achieve the following concentrations in the samples: 0.15, 0.30, and 0.60 µg/mL. Control samples contained PBS instead of escin. The samples prepared in this way were incubated for 24 h at 37 °C. At the end of incubation, cells were washed with PBS and used for further research.

Plasma membranes were extracted using the method described by Dodge et al., and the concentrations of membrane proteins were measured using the Folin–Ciocalteu assay, with the absorbance read at 750 nm [42,43].

### 4.3. Determination of the Degree of Hemolysis 

After incubation, erythrocytes were suspended in a 155 mM NaCl solution [44]. In parallel, samples of erythrocytes were prepared in distilled water, in which 100% hemolysis occurred. The prepared samples were incubated for 30 min at room temperature and then centrifuged for 5 min at 3000 rpm. The absorbance of the supernatant was measured at a wavelength of 540 nm, corresponding to the absorption maximum of hemoglobin. The obtained research results are presented in %.

### 4.4. Determination of Cell Membrane Fluidity

The fluidity of the whole RBC membrane lipids was assessed at depths of the 5th, 12th, and 16th carbon atoms in the hydrocarbon chain of fatty acids in the outer monolayer of the cell membrane. The study was conducted using the electron paramagnetic resonance (EPR) technique with the application of doxyl spin (DS) probes (5DS, 12DS, and 16DS). Spin probes were added to the erythrocytes, and then, the EPR spectrum was plotted, from which the heights of the low-field (h_+1_) and central lines (h_0_) were read. The ratio h_+1_/h_0_ was used to assess changes in erythrocyte membrane fluidity. EPS spectra were recorded using a Bruker ESP 300 E X-band spectrometer (Rheinstetten, Germany, microwave frequency of 9.73 GHz). The instrumental settings were as follows: center field: 3480 G; scan range: 80 G; modulation frequency: 100 kHz; modulation amplitude: 1 G.

### 4.5. Determination of the Conformational State of Membrane Proteins

Conformational changes in membrane proteins were assessed using the EPR technique with two spin labels (MSL and ISL) binding to free thiol groups. Isolated cell membranes were incubated for 1 h at 4 °C with spin labels. Next, the excess unbound label was removed through successive washing with a 5 mM phosphate buffer at pH 7.4 until the EPR signal disappeared in solution. For such prepared samples, the EPR spectrum was plotted, from which the heights of the low-field line (h_+1_) and central lines (h_0_) were read, and then, their ratio was calculated (h_+1_/h_0_). The obtained research results were presented as mean ± SD.

### 4.6. Measurement of the Concentration of Free Thiol Groups

The concentration of free thiols in the membrane proteins of erythrocytes was determined using 5,5′-dithiobis (2-nitrobenzoic acid) (DTNB) [45]. When DTNB interacts with free thiol groups, it produces 2-nitro-5-thiobenzoate (NTB), which has optical activity at 412 nm. The concentration of thiol groups was estimated using standard curves created from varying concentrations of reduced glutathione (ranging from 0 to 0.5 mM) and was calculated as nanomoles per milligram of protein.

### 4.7. Measurement of the Concentration of Amino Groups

The concentration of free amino groups in erythrocyte plasma membrane proteins was estimated using 2,4,6-trinitrobenzene sulfonic acid (TNBS) [46]. TNBS reacts with amines to produce a colored product that can be easily measured at a wavelength of 335 nm. The concentration of amino groups was determined based on a standard curve prepared from various concentrations of homocysteine, ranging from 0 to 300 µM. The results were expressed as nanomoles of amino groups per milligram of protein.

### 4.8. Measurement of Carbonyl Group Concentration

The protein carbonyl content in RBC plasma membranes was determined using 2,4-dinitrophenylhydrazine (DNPH) [47]. The reaction of protein carbonyl groups with DNPH produces optically active dinitrophenylhydrazones at 360 nm. The concentration of carbonyl was calculated based on the millimolar absorption coefficient of 22 mmol^−1^ cm^−1^ and expressed as nmol per milligram of protein.

### 4.9. Statistical Analysis

The normality of data was tested using the Shapiro–Wilk test. All data showed conformity with a normal distribution, so the graphs were presented as mean with bars (min to max) and raw data. The homogeneity of the variance was verified using the Mauchly sphericity test. The significance of differences between the groups was estimated using a one-way ANOVA for repeated measures and Tukey’s post hoc multiple comparison test. Statistical significance was accepted at *p* < 0.05. The statistical analysis was carried out for 6–11 independent erythrocyte samples, each in triplicate. All statistical analyses were conducted in STATISTICA. PL v.13.3.

## 5. Conclusions

In our work, we investigated red blood cell damage caused by escin. The concentrations of escin used led to damage to the plasma membrane, resulting in RBC hemolysis and increased fluidity of membrane lipids. Membrane disintegration exposed amino groups in membrane proteins but did not disrupt the membrane cytoskeleton or the structure of surface proteins. Likely, higher escin concentrations could also induce changes in the structure of membrane proteins, but this disrupted the membrane structure to a much greater extent, making measurement of its fluidity impossible. Furthermore, our studies indicate that in the treatment of chronic venous insufficiency, varicose veins, and lower limb ulcers, the maximum doses of escin should not be exceeded, as this could lead to intravascular hemolysis and oxidative stress initiated by released hemoglobin.

## Data Availability

The original contributions presented in this study are included in the article. Further inquiries can be directed to the corresponding author.
